# Importation trends in antibiotics for veterinary use in Rwanda: A retrospective study between 2019 and 2021

**DOI:** 10.1371/journal.pone.0299917

**Published:** 2024-03-07

**Authors:** Rosine Manishimwe, Balthazar Ndayisenga, Richard Habimana, Ivan Emile Mwikarago, Theobald Habiyaremye, Jean Paul Ndindibije, Anselme Shyaka, Joseph Kabatende, Vedaste Habyalimana, Charles Karangwa, Emile Bienvenu

**Affiliations:** 1 Rwanda Food and Drugs Authority, Kigali, Rwanda; 2 Center for One Health, University of Global Health Equity, Butaro, Rwanda; 3 World Health Organization, Regional Office for Africa, Brazzaville, Republic of the Congo; 4 Rwanda Forensic Institute, Kigali, Rwanda; BOKU: Universitat fur Bodenkultur Wien, AUSTRIA

## Abstract

Estimating antibiotic consumption in animals is fundamental to guiding decision-making and research on controlling the emergence and spread of antibiotic-resistant bacteria in humans, animals, and the environment. This study aimed to establish importation trends of antibiotics for veterinary use in Rwanda between 2019 and 2021. Data was collected from the Rwanda Food and Drugs Authority’s database. Quantities of imported antibiotic active ingredients were computed using the information extracted from the issued import licenses. These quantities were subsequently adjusted per animal biomass. In total, 35,291.4 kg of antibiotics were imported into Rwanda between 2019 and 2021, with an annual mean of 11,763.8 ± 1,486.9 kg. The adjustment of imported quantities of antibiotics per animal biomass revealed that 29.1 mg/kg, 24.3 mg/kg, and 30.3 mg/kg were imported in 2019, 2020, and 2021 respectively. A slight but not statistically significant decline in antibiotic importation was noted in 2020 (*p-value* = 0.547). Most of the imported antibiotics were indicated to be used in food-producing animals (35,253.8 kg or 99.9% of the imported antibiotics). Tetracyclines (17,768.6 kg or 50.3%), followed by sulfonamides (7,865.0 kg or 22.3%) and aminoglycosides (4,071.1 kg or 11.5%), were the most imported antibiotics over the studied period. It was noted that 78.9% of the imported antibiotics were categorized as highly important antimicrobials for human medicine. This study established a generalized overview of the importation of antibiotics for veterinary use in Rwanda. These results can serve as guidance for the control of antibiotic misuse. They can be used to make a correlation between antibiotic importation, antibiotic consumption, and the occurrence of antibiotic resistance in the country.

## Introduction

Antibiotics have made substantial contributions to the management of infectious diseases in humans and animals since their discovery in the twentieth century [1]. Unfortunately, the number of disease-causing agents (pathogens) resistant to common antibiotics has not stopped increasing [2]. Antibiotic-resistant bacteria have emerged and spread worldwide, posing a global danger to controlling infectious diseases in humans and animals [3]. The World Health Organization (WHO) describes antimicrobial resistance (AMR) as a phenomenon that happens when antimicrobials, such as antibiotics, become ineffective against pathogenic microorganisms like bacteria, whereby infection persists in the body [4]. According to the world bank, if no action is taken, the world will lose 3.8% of its annual Gross Domestic Product, with millions of deaths every year by 2050, and low-income countries will suffer the most [2]. Irrational use of antibiotics is cited among significant factors in the selection and emergence of antibiotic-resistant bacteria [5–8]. Hence, the control and prudent use of antibiotics should be encouraged to decrease the occurrence and emergence of antibiotic resistance. In different countries, the promotion of careful use of antibiotics in animals has been linked to a decrease in the emergence of antibiotic-resistant bacteria [9–11]. In Rwanda, the Rwanda Food and Drugs Authority (Rwanda FDA) oversees the regulation of veterinary medicines, such as antibiotics, including their importation, manufacturing, distribution, and sale as per Law No. 003/2018 of 09/02/2018, which establishes the Rwanda FDA and determines its mission, organization, and functioning [12].

In 2018, the report for a joint external evaluation of International Health Regulations (IHR) core capacities of Rwanda conducted by WHO identified antimicrobial resistance as a major public health problem with high levels of inappropriate use of antimicrobials in the human and animal sectors [13].

Like many other African countries, Rwanda has developed and endorsed a national action plan on antimicrobial resistance in line with the global action plan on AMR promoted by the quadripartite of the Food and Agriculture Organization of the United Nations (FAO), the World Organization for Animal Health (WOAH), the WHO, and the United Nations Environment Programme (UNEP) [4]. The Rwandan national action plan on antimicrobial resistance has five strategic objectives with a common goal of ensuring the ability to treat and prevent infectious diseases with quality, effective, and safe antimicrobials medicines. Specifically, the fourth strategic objective aims to optimize the use of antimicrobial agents in human and animal health by tracking antimicrobial consumption and promoting the prudent use of these molecules to preserve their sustainable efficacy [14]. The national action plan was approved in 2021. However, its implementation is still in its early stage. At present, technical working teams are being established to focus on each of the strategic objectives and the guiding documents are under development. Besides, although some studies have highlighted a high occurrence of antibiotic-resistant bacteria in humans [15–18] and animals [19, 20], no comprehensive studies have been conducted to estimate the consumption or importation of antibiotics for veterinary use in Rwanda. Baseline information on quantities of imported antibiotics for veterinary use is essential for guiding decision-making on developing specific, objective, and evidence-based interventions, to promote the prudent use of antibiotics in animals, control the emergence and spread of antibiotic-resistant bacteria, and estimate antibiotic consumption trends in a country [21].

This study was retrospectively conducted to gather information on the national importation of antibiotics for veterinary use in Rwanda to generate the empirical evidence needed for the development, implementation, and evaluation of novel ways to optimize the use of antibiotics in animals.

## Materials and methods

### Source of data

Retrospective data on imported veterinary medicinal products in Rwanda were collected from the Rwanda FDA database, in the food and drugs import-export control division. Approval to access and use data from the Rwanda FDA was obtained from the office of the director general prior to data collection (June 21^st^, 2022). A total of 1,574 import permits issued by Rwanda FDA between January 2019 and December 2021 were screened to extract information on imported veterinary medicinal products. The date of importation, the product’s brand name, the active pharmaceutical ingredients (APIs), the pack size, and the number of imported products were extracted from import permits. Additional information such as target species, the strength of APIs, the route of administration, and the dosage form was obtained from the Rwanda FDA list of authorized veterinary medicinal products [22], summaries of product characteristics, and manufacturers’ product catalogs. Veterinary medicinal products are any product with approved claims to having a prophylactic, therapeutic or diagnostic effect or to alter physiological functions when administered or applied to an animal [23] while APIs are the active components in a pharmaceutical drug that produce pharmacological activity or other direct effect in the diagnosis, cure, mitigation, treatment, or prevention of disease, or to affect the structure or any function of the body [24]. Antibiotic API is the active ingredient in an antibiotic medicine.

### Estimation of animal biomass and adjustment of antibiotic quantities by animal biomass

To compare quantities of imported antibiotics for animal use in Rwanda and quantities of imported antibiotics in other countries, the imported amounts of antibiotics were adjusted to the total animal biomass. Animal biomass is defined as the total weight of live domestic animals in a given area and year that is used as a proxy to represent animals that are likely exposed to the amounts of antibiotics reported [23]. The animal biomass was calculated following the method described by the WOAH [23]. For that, data on the census population of animals, quantity of meat production/year, and heads of animals slaughtered/year were retrieved from the FAOSTAT database [25]. The WOAH methodology was chosen as it reported to be the best for biomass estimation for global monitoring of antimicrobial sales for use in food animals [26].

A focus was placed on antibiotics indicated for use in food-producing animals, such as cattle, sheep, goats, pigs, and chickens, as the majority of antibiotics imported into Rwanda were intended for use in food-producing animals in contrast to antibiotics intended for use in companion animals such as dogs and cats. Only cattle, sheep, goats, pigs, and chickens were included in this study. Poultry was narrowed to chicken as they represent most of the poultry species reared in Rwanda. Rabbits were excluded because antibiotics intended to be used in rabbits in Rwanda are scanty [25].

### Data analysis

Data was compiled in Microsoft Excel 2016 (Microsoft Corporation, Redmond, Washington, USA). Only quantities of antibiotic were considered in this study.

The quantity of antibiotic APIs in each product was calculated in milligrams (mg) by multiplying the strength of the API by the pack size of the products and by the total number of imported products. Where strengths were given in International Unit (IU), % w/w, or % w/v, the latter were converted into mg using the conversion factors as per the WOAH’s guidelines [23]. The total quantities of imported antibiotic APIs obtained in mg were finally converted into kilograms (kg).

Adjustment of antibiotic quantities by animal biomass was made using the formulas below.


$$\text{Carcass}\;\text{weight}(\text{kg})=\frac{\text{Weight}\;\text{of}\;\text{species}\;\text{slaughtered}\;(\text{kg})}{\text{Number}\;\text{of}\;\text{species}\;\text{slaughtered}\;(\text{Heads})}$$

$$\text{Live}\;\text{weight}\;(\text{Kg})=\frac{\text{Carcass}\;\text{weight}\;(\text{kg})}{\text{Conversion}\;\text{coefficient}\;\text{K}}$$

Conversion coefficients K were extracted from the Eurostat estimates [27].


Animalbiomass(kg)=Liveweight(kg)×censusanimalpopulation

Totalanimalbiomass=cattlebiomass+goatsbiomass+pigsbiomass+chickensbiomass

To calculate the quantities of antibiotic APIs imported in Rwanda adjusted to the total animal biomass, the formula below was used.


$$\text{Adjusted}\;\text{quantitative}\;\text{data}\;\text{on}\;\text{imported}\;\text{antibiotic}\;\text{APIs}=\frac{\text{Quantity}\;\text{of}\;\text{antibiotic}\;\text{API}\;(\text{mg})}{\;\text{Total}\;\text{animal}\;\text{biomass}\;(\text{kg})}$$

Imported antibiotic APIs were grouped into antibiotic classes based on the WHO Anatomical Therapeutic and Chemical for veterinary medicines system (WHO ATCvet) [28]. Furthermore, imported antibiotic APIs were classified based on the WHO levels of importance of antimicrobials for human medicine [29]. The four levels of importance of antimicrobials are:

- Critically important antibiotics such as aminoglycosides, 3^rd^,4^th^, and 5^th^ generation of cephalosporins, polymyxins, penicillins (aminopenicillins, aminopenicillin with beta-lactamase inhibitors), and quinolones.
- Highly important antibiotics such as penicillins (narrow spectrum), 1^st^ and 2^nd^ generation of cephalosporins, sulfonamides, dihydrofolate reductase inhibitors and combinations, and tetracyclines.
- Important antibiotics such as aminocyclitols, cyclic polypeptide, nitrofuran derivatives and nitroimidazoles.
- Not classified antibiotics.

Descriptive statistics such as proportions and averages were performed using Microsoft Excel 2016. R 4.1.3 (R Foundation, Boston, Massachusetts, USA) was used to perform the Kruskal–Wallis test to compare the quantities of imported antibiotic APIs between years of importation (a nonparametric test was used as data were not normally distributed). Statistical significance was defined as a *p-value* of less than 0.05.

## Results

According to the 1,574 permits issued between 2019 and 2021 to import veterinary medicinal products into Rwanda, anthelmintic products were the most frequently veterinary medicinal products imported, followed by antibiotics, while antipsychotics were rarely imported during this period (Table 1).

**Table 1:** Frequencies of imported veterinary medicinal products per year of importation.

	Number of import permits issued for veterinary medicinal products				
Category of veterinary medicinal products	2019	2020	2021	Total	%
Anthelmintic	136	210	146	492	31.3
Antibiotics	147	138	168	453	28.8
Ectoparasiticides	46	53	37	136	8.6
Antiprotozoal	36	43	46	125	7.9
Vitamin supplements	33	33	28	94	6.0
Hormones	36	6	16	58	3.7
Endectocide	13	14	24	51	3.2
Anti-inflammatory	12	14	17	43	2.7
Analgesics	7	7	21	35	2.2
Mineral supplements	8	13	11	32	2.0
Corticosteroids	4	8	11	23	1.5
Minerals and vitamins supplements	5	4	4	13	0.8
Hypnotics and sedatives	1	2	3	6	0.4
Anesthetics	0	3	1	4	0.3
Antihistamines	1	0	2	3	0.2
Diuretics and Antioxidants	1	1	0	2	0.1
Herbal oil	0	1	1	2	0.1
Anticholinergics	0	1	0	1	0.1
Antipsychotics	0	0	1	1	0.1
**Total**	**486**	**551**	**537**	**1574**	**100.0**

### Antibiotics for veterinary use imported into Rwanda between 2019 and 2021

**Fig 1:**
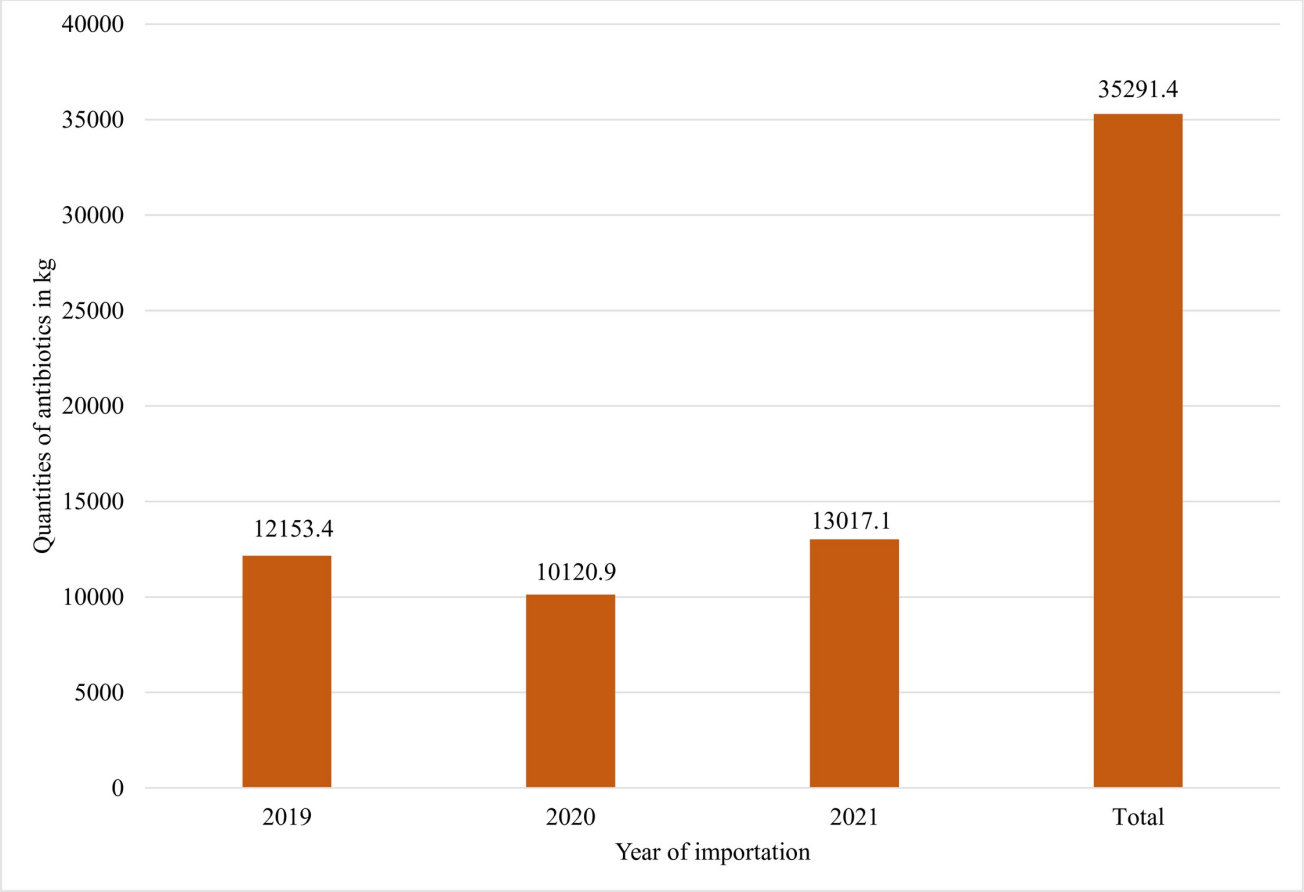
Quantities of imported antibiotics (kg of active pharmaceutical ingredients) by year of importation.

In total, 453 antibiotics, equivalent to 35,291.4 kg, were imported between 2019 and 2021 (Fig 1). The annual mean of imported antibiotics was 11,763.8 ± 1,486.9 kg. The importation of antibiotic was found not statistically different between the three years of importation (*p-value* = 0.547).

The majority of imported antibiotics were intended to be used in food-producing animals (*p-value* < 0.001) through the parenteral route of administration (*p-value* < 0.001), as presented in Table 2.

**Table 2:** Imported antibiotics (kg of active pharmaceutical ingredients) per target animal and route of administration.

Route of administration (p-value<0.05)	kg of APIs (p-value<0.05)				
	Antibiotics for both companion and food producing Animals	Antibiotics for companion animals only	Antibiotics for food-producing animals only	Total	%
Parenteral	3.5	0.0	18,912.6	**18,916.1**	**53.6**
Oral	0.0	2.0	16,207.1	**16,209.2**	**45.9**
Other routes	0.0	0.0	84.6	**84.6**	**0.2**
Topical	32.1	0.0	49.4	**81.6**	**0.2**
**Total**	**35.6**	**2.0**	**35,253.8**	**35,291.4**	**100.0**
**%**	**0.10**	**0.0**	**99.9**	**100.0**	

Companion animals include dogs and cats. Food-producing animals encompass cattle, sheep, goats, pigs, and chickens. Companion animals and food-producing animals comprise dogs and/or cats and at least one of cattle, sheep, goats, pigs, and chickens. The first column, ’Companion and Food-Producing Animals,’ represents quantities of imported antibiotic APIs that can be used in both companion and food-producing animals. Other routes of administration include among others intramammary route and intrauterine route. The differences in kg of active pharmaceutical ingredients (APIs) per targeted animal and per route of administration are statistically significant.

Antibiotics for animal use imported into Rwanda were dominated by tetracyclines followed by sulfonamides, and aminoglycosides (Table 3).

**Table 3:** Quantities of imported antibiotics by antibiotic class and year importation.

			Quantities of antibiotic-active pharmaceutical ingredients imported in kg					
Class of Antibiotics	Antibiotic API	ATCvet Code	2019	2020	2021	Total quantity	Total quantity by Class	%
Tetracyclines	Oxytetracycline	QJ01AA06	5,321.1	5,201.4	6,628.6	17,151.1	17,768.6	50.3
	Tetracycline	QJ01AA07	4.0	28.0	30.5	62.5		
	Doxycycline	QJ01AA02	249.3	102.8	202.9	555.0		
Sulfonamides	Sulfadimidine	QJ01EQ03	2,717.4	2,530.7	2,449.2	7,697.3	7,865.0	22.3
	Sulfadoxine	QJ01EQ13	21.1	38.4	67.1	126.6		
	Sulfamethoxazole	QJ01EQ11	12.0	7.2	13.0	32.2		
	Sulfadiazine	QJ01EQ10	0.0	2.9	6.0	8.9		
Aminoglycosides	Dihydrostreptomycin	QJ01GA90	1,472.7	800.0	1,326.7	3,599.4	4,071.1	11.5
	Streptomycin	QJ01GA01	34.1	21.0	216.0	272.1		
	Gentamicin	QJ01GB03	9.6	21.7	83.1	114.4		
	Neomycin	J01GB05	55.4	19.9	9.9	85.2		
Beta-Lactams	Penicillin G	QJ01CE01	890.8	483.8	912.2	2,286.8	2,779.5	7.9
	Amoxicillin	QJ01CA04	164.1	106.4	191.4	461.9		
	Amoxicillin- Clavulanic acid	QJ01CR02	0.0	1.2	0.8	2.0		
	Ampicillin	QJ01CA01	0.0	0.2	0.0	0.2		
	Cloxacillin	QJ01CF02	7.3	12.8	8.4	28.5		
Macrolides	Tylosin	QJ01FA90	648.0	221.2	334.1	1,203.4	1,296.1	3.7
	Erythromycin	QJ01FA01	33.6	21.0	37.8	92.4		
	Spiramycin	QJ01FA02	0.0	0.4	0.0	0.4		
Polypeptides	Colistin	QJ01XB01	340.9	89.4	335.9	766.1	766.1	2.2
Fluoroquinolones	Enrofloxacin	QJ01MA90	90.2	169.9	96.3	356.4	356.4	1.0
Nitrofurans	Furazolidone	QJ01XE90	75.0	225.0	50.0	350.0	350.0	1.0
Trimethoprim	Trimethoprim	QJ01EA01	6.6	9.7	17.2	33.5	33.5	0.1
Pleuromutilins	Tiamulin	QJ01XQ01	0.0	5.0	0.0	5.0	5.0	0.0
**Total**			**12,153.4**	**10,120.9**	**13,017.1**	**35,291.4**	**35,291.4**	100.0
**%**			34.4	28.7	36.9	100.0	100.0	

ATCVet: Anatomical therapeutic chemical classification system for veterinary medicine

API: Active pharmaceutical ingredient

**Fig 2:**
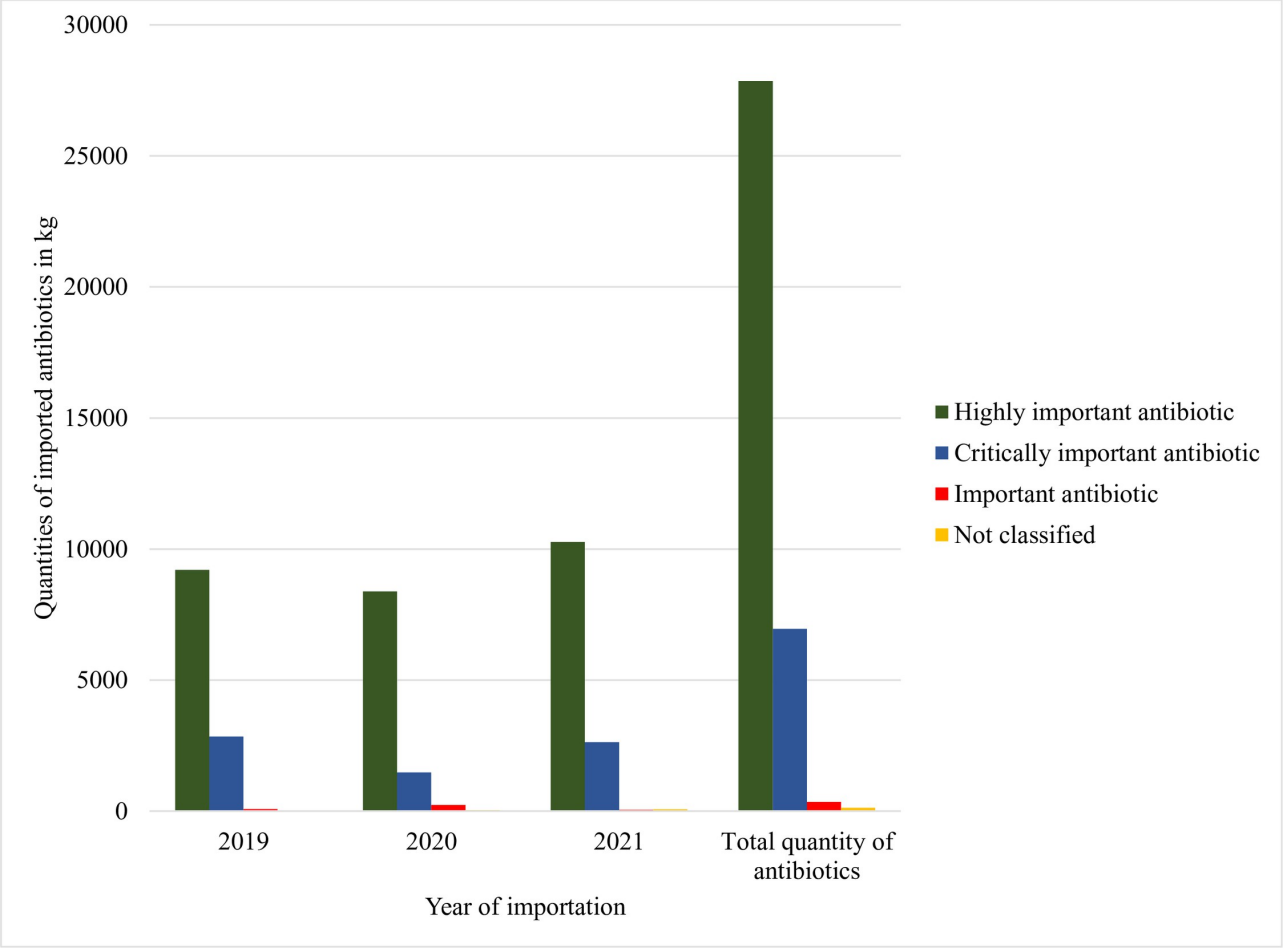
Imported antibiotics according to the World Health Organization levels of importance of antimicrobials for human medicine. APIs: Active Pharmaceutical Ingredients.

The results show that 78.9% of the imported antibiotics for animal use are classified as highly important for human medicine while 19.7% are critically important antibiotics for human medicine (Fig 2).

### Adjustment of quantities of imported antibiotics by the total animal biomass

**Fig 3:**
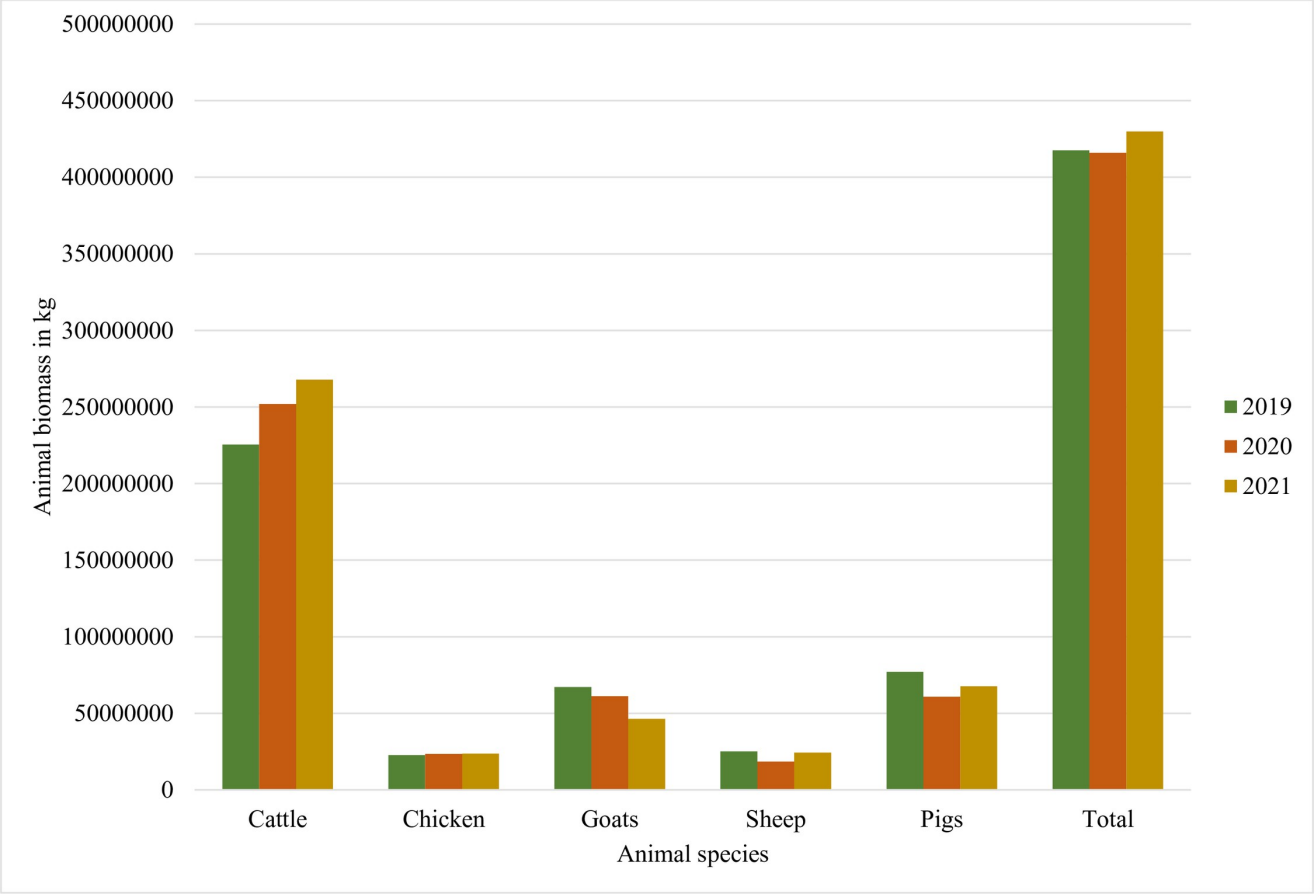
Animal biomass per animal species in 2019, 2020, and 2021 in Rwanda.

The total animal biomass in 2019, 2020, and 2021 was estimated to be 417,522,942.3 kg, 415,915,389.7 kg, and 429,863,472.6 kg respectively. In 2019, 2020, and 2021 the total animal biomass was dominated by cattle (Fig 3).

The adjustment of quantities of antibiotics for food-producing animals imported into Rwanda showed that the mg of antibiotics per kg of animals slightly decreased between 2019 and 2020 to increase again in 2021. The Table 4 presents the quantities of antibiotics imported in Rwanda adjusted by total animal biomass.

**Table 4:** Quantities of antibiotics imported into Rwanda to be used in food-producing animals adjusted by total animal biomass.

	2019	2020	2021
**Quantity of imported antibiotics in mg**	12,153,397,960	10,119,670,530	13,016,294,160
**Total animal biomass in kg**	417,522,942.3	415,915,389.7	429,863,472.6
**Adjusted quantities of antibiotics in mg/kg**	29.1	24.3	30.3

## Discussion

This study aimed to establish trends in the importation of antibiotics for veterinary use in Rwanda. With no pharmaceutical industries for veterinary antibiotic manufacturing in the country, all antibiotics used in animals in Rwanda are solely imported. Hence, the results of this study reliably portray the consumption of most antibiotics for veterinary use in Rwanda.

It was noted that anthelmintic and antibiotics are the major veterinary medicinal products imported and this may inform on the animal diseases most frequently encountered in animals in the country (parasitic and bacterial infections). Indeed, bacterial and parasitic infections are reported as common diseases in livestock in Rwanda [30]. Disease control strategies, such as vaccination campaigns and promotion of biosecurity measures in farms, should be emphasized to minimize the use of these veterinary medicinal products and preserve their efficacy.

In contrary to the situation in Cameroon [31], most of the imported antibiotics into Rwanda were indicated to be used via the parenteral route of administration. This implies that most imported antibiotics were for systemic treatment that leads to a distribution of antibiotics in muscle, milk, and other consumed parts of an animal. Residues of antibiotics in food products can be anticipated. Antibiotic residues in food for human consumption are a public health concern, as these residues at a sub-therapeutic dose can promote the selection and emergence of antibiotic-resistant bacteria in humans [32]. Therefore, it is crucial to emphasize awareness campaigns to sensitize the respect for the withdrawal period in food-producing animals.

A difference between quantities of antibiotics for veterinary use imported into Rwanda and quantities imported in other countries in the region was noted. Quantities of antibiotics for veterinary use imported into Rwanda are relatively small compared to quantities of antibiotics imported in Tanzania and Kenya, even though these two countries have local manufacturers of veterinary medicinal products [33, 34]. In Tanzania, it was reported that between 2010 and 2017, 12,147,491.5 kg of antibiotics were imported, with an annual mean of 1,518,436.4 kg of antibiotics for veterinary use [35]. In Kenya, the yearly mean of imported antimicrobials for veterinary use was estimated to be 14,593.7 kg between 1995 and 1999 [36]. Besides, in Cameroon, on average, 36,280 kg of antibiotics are imported annually to be used in food-producing animals [31]. In South Africa, between 2018 and 2020 there was a 28.6% increase in antibiotics imported for animals to 2,48 tons with 1,970,241 kg, 2,398,521 kg, and 2,488,754 kg of antibiotics imported in 2018, 2019, and 2020 respectively [37]. Although quantities of antibiotics for veterinary use imported in Rwanda appear to be different from quantities of antibiotics imported in other countries, conclusive comparison can’t be drawn as some of these countries have local manufacturers of veterinary medicinal products. Hence their national antibiotic consumption doesn’t rely on importation only. In addition, antibiotic consumption is proportionate to a country’s animal population [38]. In contrast to Tanzania and Kenya, Rwanda’s animal population is relatively small [39]. For instance, Tanzania and Kenya are among the countries with the largest cattle population in sub-Saharan African countries [40]. Hence, adjusted quantities by animal biomass should be used to make conclusive comparisons.

Quantities of antibiotics adjusted by the total animal biomass in Rwanda were not different from the data reported by the WOAH for the Africa region [23]. The WOAH fifth report on antimicrobial agents used in animals showed that for the 24 African countries that participated in the data collection, the estimated total animal biomass was dominated by cattle followed by sheep and goats [23]. In this study, the total biomass was dominated by cattle, followed by goats, pigs, and sheep.

The WOAH report states that for the African region, the number of antibiotics consumed adjusted by animal biomass ranged between 32.7 mg/kg and 25.9 mg/kg between 2014 and 2017 [23]. Results obtained from this study (29.1 mg/kg, 24.3 mg/kg, and 30.3 mg/kg) are not far from the data reported by WOAH. However, these results remain very low compared to data reported in other regions of the world, where adjusted antibiotics quantities varied between 57.4 mg/kg in Europe and 192.2 mg/kg in Asia [23]. In 2015, Van Boeckel and collaborators reported that in Africa, the only remarkable spots of consumption of antimicrobials in food-producing animals were in the Nile Delta and South Africa [41].

In 2017, it was estimated that the African consumption of antimicrobials for food animals would increase by 37% in 2030 [42]. Hence, surveillance of antimicrobial consumption is encouraged to control these medicinal products’ overuse.

According to our findings there was a slight decline in the importation of antibiotics in 2020, possibly caused by the Covid-19 pandemic that impacted many sectors, including international trade of medicines intended for animal health [43].

Most of the antibiotics imported for veterinary use were intended to be used in food-producing animals. This correlates with the report of WOAH, where most of the African countries that managed to distinguish antimicrobial quantities by animal groups provided data for antibiotics used in food animals [23]. This represents an area of vigilance as food-producing animals can be incubators and spreaders of antimicrobial-resistant bacteria if antibiotics are not rationally used in these animals [44]. In 2017, Manishimwe and collaborators reported a misuse of antibiotics in farm animals in Rwanda [45].

Tetracyclines were the most imported antibiotics for veterinary use in Rwanda. Tetracycline antibiotics remain reported as the most frequently consumed antibiotics for veterinary use in Tanzania [35], Kenya [36], Cameroon [31], in South Africa [46] and many other African countries as well as the rest of the world [23]. Tetracyclines have been proven to have additional clinical uses besides antimicrobial activities. Hence, they are reported to be widely used for prophylaxis and therapy of human and animal infections due to their multiple clinical uses and their inexpensiveness [47]. For instance, some African countries explained that tetracycline is the major class of antibiotic used in animals because of its low cost [23]. The elevated consumption of tetracycline in food animals can also be related to the high level of prevalence of bacteria resistant to tetracycline reported in different food-producing animals in Rwanda [19].

The categorization of imported antibiotics based on the WHO levels of importance of antimicrobials for human medicine [29] showed results similar to results reported in Cameroon [31]. Most imported antibiotics into Rwanda, such as tetracycline, sulfonamides, and penicillins, are classified as highly important antibiotics for human medicine. But some of the imported antibiotics, such as aminoglycoside, erythromycin, or colistin, are classified as critically important antibiotics for human medicine. This trend is also reported in other African and European countries where highly important antibiotics for human medicine, such as tetracycline and penicillin, are widely used in food-producing animals [31, 48] Cases of using critically important antibiotics for human medicine have also been reported in several African countries [31, 49]. The WHO recommended a reduction of using medically important antibiotics in food-producing animals [50]. This recommendation was well received by different stakeholders in the industry of food animals [51].

The foremost limitation of this study is that analyzed data were obtained from the national importation records, which cannot give a clear picture of how these antibiotics were used in animals with respect to the indicated dose, the duration of treatment, use in the right animal species, use of antibiotics for therapeutic, for prevention or growth promotion, and so on.

Additionally, only legally imported antibiotics were covered by the study. However, given that the country is enforcing, among other things, the implementation of high standards and efficient control of veterinary medicines on the market, some antibiotics may be imported through unauthorized circuits, whose data, if included, would change the results.

Quantities of antibiotics imported in Rwanda were not adjusted by each animal species biomass because some antibiotics are indicated to be used in more than one animal species. Hence, the quantities of antibiotics imported were adjusted by the total animal biomass of food-producing animals without any stratification in specific animal species.

### Conclusion

This study establishes a baseline of imported antibiotics for veterinary use in Rwanda. Most of the imported antibiotics are indicated for use in food-producing animals, with tetracyclines being the top class imported for veterinary use. Quantities of antibiotics imported into Rwanda, adjusted by animal biomass, are not significantly different from those reported in other African countries. This study is the first of its kind in the country to offer information on animal antibiotics importation. It forms a basis for future research, such as establishing a link between antibiotics use in animals and the occurrence of antibiotic-resistant bacteria in both animals and humans. This study generated data that will assist in the promotion of antibiotic stewardship in animals and the control of antibiotic resistance in Rwanda.

## Supporting information

S1 Data (XLSX)
